# Rupture of Rectum and Vagina in a Mare during Parturition: Successful Treatment1Read before the Fifteenth Annual Meeting of the Iowa State Veterinary Medical Association, Cedar Rapids, Iowa, January 14 and 15, 1903.

**Published:** 1903-02

**Authors:** S. H. Bauman

**Affiliations:** Birmingham, Iowa


### REPORTS OF CASES.

### RUPTURE OF RECTUM AND VAGINA IN A MARE DURING PARTURITION: SUCCESSFUL TREATMENT.[1]

By S. H. Bauman, D.V.S.,
BIRMINGHAM, IOWA.

On March 29, 1902, I was called to Keosauqua to see a fine saddle-bred mare, and was informed by the owner that the mare had been delivered of a colt through the rectum. That he had been watching the mare and went to the house for a short time, and on his return to the barn found the front legs and nose protruding from the rectum. He undertook to shove the colt back, but could do nothing. The colt was delivered in a short time and seemed all right. I arrived about five hours after the birth of the colt. The mare was uneasy and nervous, but otherwise was all right. Upon examination I found the rectovaginal septum completely ruptured. The perineum was not torn through, but the rupture extended down to the left of the vulva close to the hip. It being late, and not having a suitable place to operate, I had the mare brought to my barn next day, when I bought the mare and the colt. On examination I found the parts badly swollen. No feces had been passed since parturition, and the vagina was completely filled. After removing the contents and thoroughly cleansing the parts with a creolin solution I allowed all ragged edges to remain, not doing any trimming with the scissors or knife. I took heavy twine and tape and put in as many sutures as possible, part interrupted and others of any kind I happened to get; then packed the vagina completely full of carbolized oakum for a support to the stitches. The mare was tied so that she could not lie down. The fecal matter was all drawn by hand and the packing in the vagina changed every third day. Whenever a weakening in stitches was found a new one was inserted. In about twenty days the septum between the vagina and rectum was completely closed. My great trouble was in the external opening. The excrement would work down into the rupture in spite of all I could do, and in cleaning out the same had to break granulations almost as fast as they would form, so I commenced to remove the feces with the hand again and not allow the mare to do any straining, and in a short time the opening closed. The mare was

[1] Read before the Fifteenth Annual Meeting of the Iowa State Veterinary Medical Association, Cedar Rapids, Iowa, January 14 and 15, 1903.

turned into pasture in about six weeks, but it was August before I quit treatment. Weaned the colt in September and commenced driving the mare October 1st. The only inconvenience I find is, that the anus remains slightly open when the mare is very gaunt after a long and hard day's drive. By the way, I have one of the best drivers I ever owned. The filly is a splendid and very promising one. Owing to the nature of the food and the fasting, the mare did not furnish sufficient nurse for the colt, so we had to feed cow's milk, which I diluted with water and added lime-water, sugar, and brandy.

